# Publisher Correction: Fuelling conditions at staging sites can mitigate Arctic warming effects in a migratory bird

**DOI:** 10.1038/s41467-018-07408-2

**Published:** 2018-11-30

**Authors:** Eldar Rakhimberdiev, Sjoerd Duijns, Julia Karagicheva, Cornelis J. Camphuysen, VRS Castricum, Anne Dekinga, Rob Dekker, Anatoly Gavrilov, Job ten Horn, Joop Jukema, Anatoly Saveliev, Mikhail Soloviev, T. Lee Tibbitts, Jan A. van Gils, Theunis Piersma

**Affiliations:** 1 NIOZ Royal Netherlands Institute for Sea Research, Department of Coastal Systems and Utrecht University, PO Box 59, 1790 AB Den Burg, Texel The Netherlands; 2 Department of Vertebrate Zoology, Biological Faculty, Lomonosov Moscow State University, 119991 Moscow, Russia; 3 Department of Biology, Carleton University, 1125 Colonel By Drive, K1S 5B6 Ottawa, ON Canada; 4 Directorate of Taimyrsky Reserves, 663305 Norilsk, Russia; 5 Haerdawei 62, 8854 AC Oosterbierum, The Netherlands; 6 Institute of Environmental Sciences, Kazan Federal University, 420097 Kazan, Russia; 7 U.S. Geological Survey Alaska Science Center, 4210 University Drive, Anchorage, AK 99508 USA; 8 Chair in Global Flyway Ecology, Conservation Ecology Group, Groningen Institute for Evolutionary Life Sciences (GELIFES), University of Groningen, PO Box 11103, 9700 CC Groningen, The Netherlands

Correction to: *Nature Communications* 10.1038/s41467-018-06673-5; published online 15 October 2018.

In the original HTML version of this Article, the order of authors within the author list was incorrect. The consortium VRS Castricum was incorrectly listed after Theunis Piersma and should have been listed after Cornelis J. Camphuysen. This error has been corrected in the HTML version of the Article; the PDF version was correct at the time of publication.

