# Supplementary material for: Fuelling conditions at staging sites can mitigate Arctic warming effects in a migratory bird
Source: Nat Commun. 2018 Oct 15;9:4263. doi: 10.1038/s41467-018-06673-5 (PMC6189115; doi:10.1038/s41467-018-06673-5)
Supplement: Supplementary file 1 — Supplementary Information [file 41467_2018_6673_MOESM1_ESM.pdf]

Supplementary Information for

Fuelling conditions at staging sites can mitigate Arctic warming effects in a migratory bird

Eldar Rakhimberdiev*, Sjoerd Duijns, Julia Karagicheva, Cornelis J. Camphuysen, VRS Castricum, Anne Dekinga, Rob Dekker, Anatoly Gavrilov, Job ten Horn, Joop Jukema, Anatoly Saveliev, Mikhail Soloviev, T. Lee Tibbitts, Jan A. van Gils, Theunis Piersma.

correspondence to: eldarrak@gmail.com

This PDF file includes:

- Supplementary Figure 1 to 2
- Supplementary Table 1 to 2

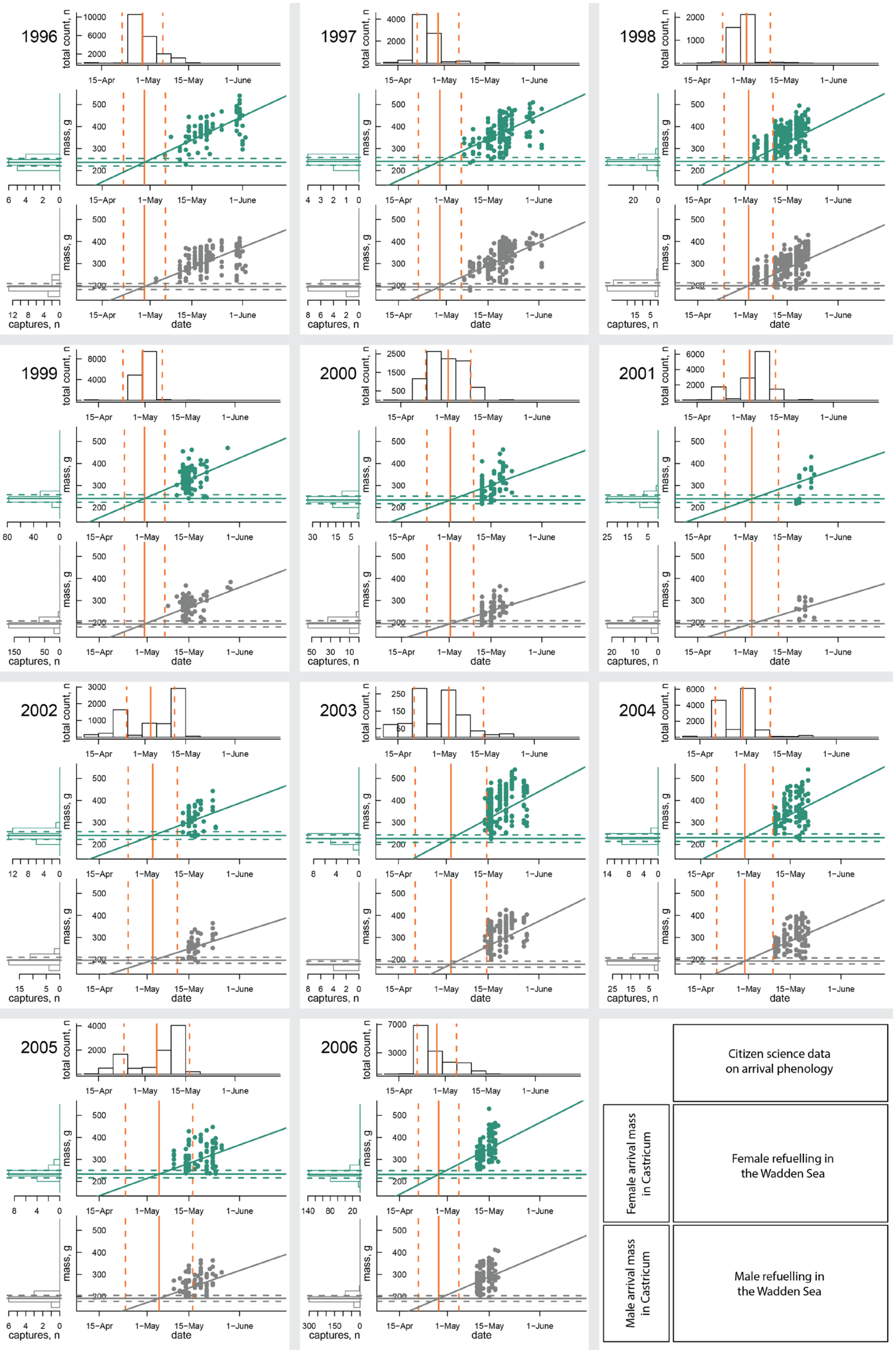

Supplementary Figure 1. Raw data and modelled results for citizen science arrival dates in the Netherlands (trektellen.nl), sex-specific arrival mass in captures in Castricum and refuelling in the Wadden Sea for 1996 – 2006. See Fig. 3 for more details.

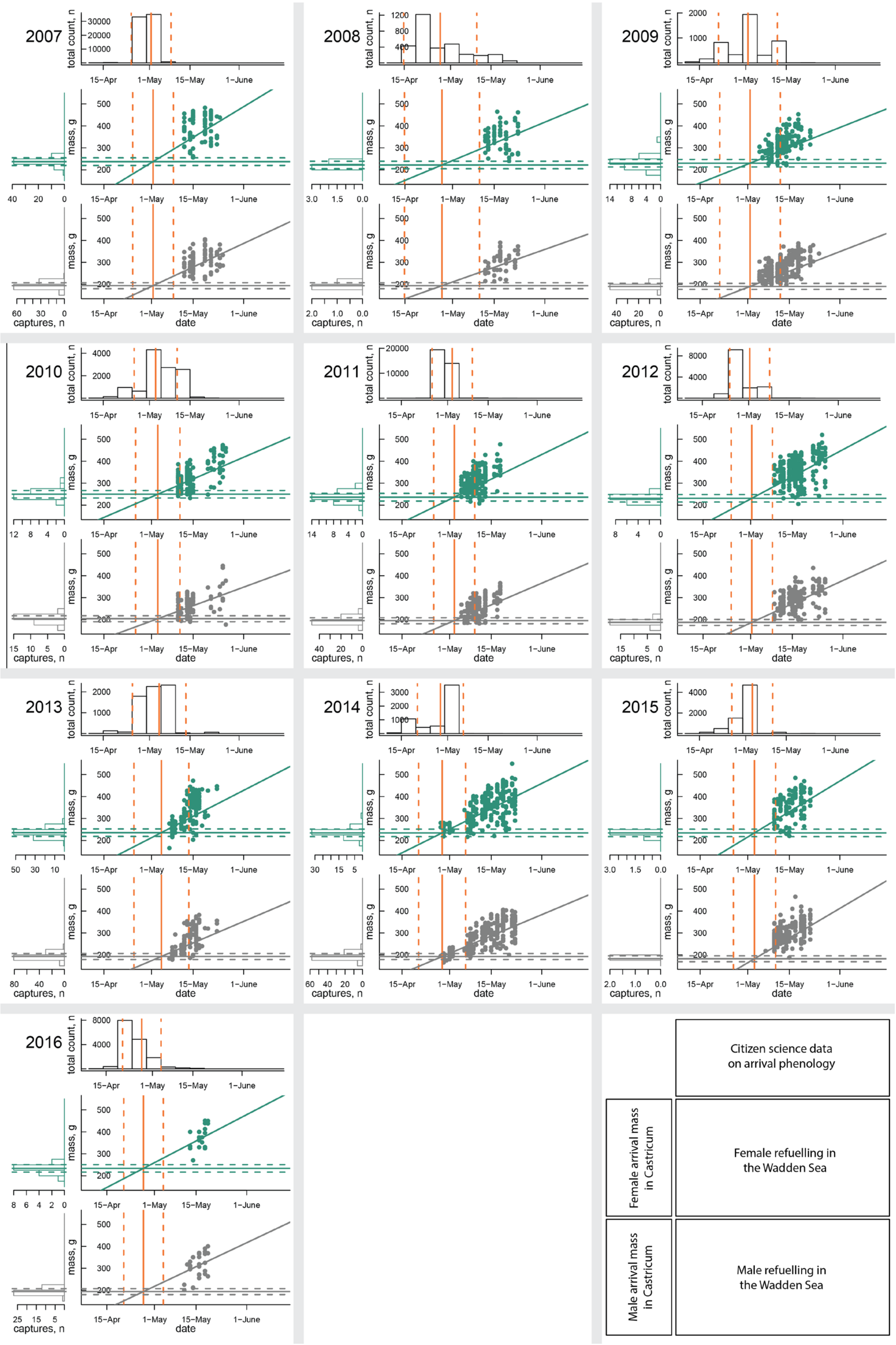

Supplementary Figure 2. Raw data and modelled results for citizen science arrival dates in the Netherlands (trektellen.nl), sex-specific arrival mass in captures in Castricum and refuelling in the Wadden Sea for 2007 – 2016. See Fig. 3 for more details.

Supplementary Table 1. *Limosa lapponica taymyrensis* breeding grounds phenology. Snowmelt represents date of snowmelt measured at the Southern Taimyr field site (72.8°N, 106.0°E). Arrival dates are obtained for Khatanga village region (72.0°N 102.5°E) and the appearance of crane-flies and clutch initiation dates were observed at southern Taimyr (72.8°N, 106.0°E).

Year	Snowmelt	First arrival to the breeding grounds	First crane-fly appearance	Clutch initiation
1992		11-Jun-92		
1993		05-Jun-93		
1994	22-Jun-94		06-Jul-94	25-Jun-94; 02-Jul-94
1995	07-Jun-95		28-Jun-95	14-Jun-95
1996	27-Jun-96	04-Jun-96	07-Jul-96	27-Jun-96
1997	06-Jun-97	01-Jun-97		
1998	13-Jun-98	01-Jun-98	30-Jun-98	
1999	04-Jun-99		29-Jun-99	11-Jun-99
2000	14-Jun-00	02-Jun-00	06-Jul-00	14-Jun-00
2001	06-Jun-01	01-Jun-01	25-Jun-01	18-Jun-01
2002	11-Jun-02	29-May-02	22-Jun-02	11-Jun-02
2003	16-Jun-03	04-Jun-03	26-Jun-03	
2004		07-Jun-04		
2005		31-May-05		
2006		01-Jun-06		
2007		28-May-07		
2008	19-Jun-08	02-Jun-08	07-Jul-08	
2009	10-Jun-09	30-May-09	27-Jun-09	11-Jun-09
2010	25-May-10	02-Jun-10	23-Jun-10	13-Jun-10; 22-Jun-10
2011	30-May-11	29-May-11	29-Jun-11	07-Jun-11; 10-Jun-11
2012	03-Jun-12	30-May-12	21-Jun-12	
2013	02-Jun-13	26-May-13	02-Jul-13	
2014	08-Jun-14	24-May-14	19-Jun-14	
2015		03-Jun-15		
2016		29-May-16		

Supplementary Table 2. Estimated coefficients from the structural equation model for bar-tailed godwit phenology

**Effect of**	**on**	**mean**	**sd**	**median**	**2.5%**	**97.5%**	***P*(|b|>0)**
Time	Arrival Wadden Sea	-0.046	0.065	-0.047	-0.170	0.084	0.77
Time	Snowmelt	-0.792	0.153	-0.793	-1.090	-0.489	1.00
Time	Arrival to Taimyr	-0.176	0.181	-0.175	-0.530	0.182	0.84
Snowmelt	Arrival to Taimyr	0.198	0.172	0.197	-0.140	0.538	0.88
Arrival Wadden Sea	Arrival to Taimyr	0.240	0.445	0.241	-0.650	1.112	0.71
Time	Crane fly emergence	-0.152	0.354	-0.150	-0.860	0.558	0.68
Snowmelt	Crane fly emergence	0.274	0.302	0.273	-0.320	0.878	0.83
Time	Clutch Initiation	0.116	0.647	0.104	-1.150	1.445	0.57
Snowmelt	Clutch Initiation	0.239	0.489	0.237	-0.760	1.226	0.71
Arrival to Taimyr	Clutch Initiation	1.604	1.239	1.667	-1.07	3.980	0.90
Crane fly emergence	Clutch Initiation	0.036	0.536	0.013	-1.01	1.176	0.51
